# Electrocardiogram in patients with acute inferior myocardial infarction due to occlusion of circumflex artery

**DOI:** 10.1097/MD.0000000000006095

**Published:** 2017-10-20

**Authors:** Qun Li, De-Zhao Wang, Bu-Xing Chen

**Affiliations:** Department of Cardiology, Beijing Tiantan Hospital, Capital Medical University, Beijing, China.

**Keywords:** acute inferior myocardial infarction, electrocardiographic, infarct-related artery, left circumflex branch

## Abstract

To investigate the diagnostic value of electrocardiographic (ECG) ST-segment in acute inferior myocardial infarction (AIMI) caused by the left circumflex branch (LCX).

A total of 240 clinical cases with AIMI in our hospital were retrospectively analyzed. All of them had received percutaneous coronary intervention (PCI) within 12 hours after symptom onset. The clinical features, ECG manifestations, and coronary artery lesion characteristics of the patients were collected.

The right coronary artery (RCA) was shown to be the infarct-related artery (IRA) in 177 patients, while LCX was responsible for AIMI in 63 cases. There was no significant difference in the risk factors of coronary heart disease (CHD) (*P* > .05 for all) between the 2 groups. ST-segment elevation in lead II, III, and AVF could be found in all patients. Moreover, ST-segment depression in lead I (STD I), ST-segment elevation in lead III (STE III), STE III-STE II, STE AVF, STD AVL, STD AVL-STD I and STE v6 lead ST-segment deviation exhibited significant difference in 2 groups (*P* < .05 for all). The changes of STD I, STE III < STEII, STD AVL < STD I could discriminate between LCX and RCA in AIMI patients with high sensitivity and specificity.

ECG may be an effective tool to predict the IRA in patient with AIMI.

## 1 Introduction

Acute myocardial infarction (AMI), commonly referred to as heart attack, is a type of coronary heart disease (CHD) that causes great mortality especially in industrialized countries.^[1,2]^ Despite of the decreased mortality caused by AMI observed in high-income countries, the low- and middle-income countries still exhibit an increasing trend.^[3,4]^ AMI is often caused by insufficient blood flow and oxygen supply to heart muscle owing to thrombosis, erosion, or rupture of the coronary atherosclerotic plaque.^[5]^ Globally, there were a total of 8.6 million MI cases in the year of 2013.^[6]^ MI can be divided into inferior, anterior, posterior, septal, and lateral. In addition, according to the ST elevation on the electrocardiogram (ECG), the myocardial infarctions (MIs) are commonly categorized into ST elevation MI (STEMI) and non-ST elevation MI (NSTEMI).^[7]^ STEMI is characterized by poor oxygenation of the heart, ST segments elevation on ECG, and increased levels of proteins associated with heart muscle's death in the blood, accounting for 25% to 40% of all cases.^[8]^ Early diagnosis and timely treatments are pivotal for outcomes of patients suffering from STEMI. Therefore, initiating monitoring of ECG is necessary for patients with STEMI to improve the management of the disease.^[9]^

The occlusion of left circumflex branch (LCX) or right coronary artery (RCA) is the major reason for acute inferior myocardial infarction (AIMI). The infarct-related artery (IRA) of AIMI can significantly influence the disease progression of AIMI patients.^[10]^ When RCA shown to be the IRA, the patients often undergo right ventricular infarction, intermittent cardiac pacing, as well as severe hemodynamic complications, leading to shock, arrhythmias, and even death.^[10–12]^ However, the prognosis for those with LCX occlusion is much better.^[10]^ Unfortunately, the RCA shown to be the culprit artery in AIMI is much more common than the LCX.^[13]^ In order to prevent and improve the adverse complications of AIMI, early identification of the IRA is required. Currently, coronary angiography has emerged as a gold standard for recognition of IRA among patients with AIMI. However, the procedures of convention coronary angiography are invasive, moreover, the method shows difficulty in the selection of ostium of anomalous vessel, due to the lack of 3D information and unclear association of coronary arteries with great vessels.^[14]^ Identification of IRA represents a great challenge for patients with AIMI.^[15]^ In the 1980s, the ECG was introduce to solve this problem.^[16]^ In the case of AIMI, there are ST elevations in leads II, III, and AVF, but the elevation level of inferior leads caused by RCA and LCX are different owing to the different vector direction of ST elevations.^[17]^ Several previous studies have demonstrated that the ST segment changes in ECG could be used to identify the IRA in AIMI.^[13,18–22]^ For example, according to the retrospective analysis of Datz et al,^[23]^ when chest lead ST segment elevated and II, III, and AVF lead ST segment elevated, the left anterior descending (LAD) branch distal occlusion could occur, and the specificity was 100%. In the study of Hertz et al,^[24]^ when inferior II lead ST-segment > III lead ST-segment and I lead ST-segment > AVL lead ST-segment, the positive predictive value of LCX occlusion was 100%.

Although changes of body surface 12-lead ECG play an important role in the diagnosis of MI and identification of IRA, it is difficult to diagnose AMI that is caused by left circumflex branch occlusion according to 12-lead ECG. This study, supported by coronary angiography, has confirmed the clinical features and ECG changes of AIMI caused by different IRA, and aims to find out the ECG manifestations of patients whose IRA is the left circumflex branch.

## 2 Material and methods

### 2.1 Study subjects

This study was approved by the Ethics Committee of Beijing Tiantan Hospital. All the patients signed the informed consents. A retrospective clinical analysis was performed for 240 AIMI patients admitted to the Department of Cardiology in Beijing Tiantan Hospital from Jan 1, 2004 to Dec 31, 2004. The study subjects included 84 men and 156 women, with the average age of 60.6 ± 11.2 years. The electrocardiographic data of the patients were available. They were divided into 2 groups (RCA group and LCX group) according to IRA. The patients collected in the current study should meet the following inclusion criteria: diagnostic criteria of ST-segment elevation myocardial infraction (STEMI): myocardial ischemic symptoms ≥20 minutes; ECG changes: new onset of 2 or 3 inferior leads including II, III, AVF; ST-segment elevation: ≥0.01 mV; cardiac markers increased over 99% of the normal value. Time from the onset of myocardial ischemia symptom to the ECG examination: ≤12 hours. The gold standard for diagnosis of IRA responsible for coronary artery diseases depended on the coronary angiogram within 12 hours. First time attack STEMI. Coronary angiogram revealed that LCX or RCA was the only coronary artery lesion and IRA. Exclusion criteria: double or multivessel diseases; old myocardial infarction; left ventricular hypertrophy; complete left bundle branch block (LBBB) or complete right bundle branch block (RBBB); diseases that affected ST-segment deviation, such as pacing rhythm, pericarditis, early repolarization syndrome (ERS), AMI, variant angina, and hyperkalemia; data of ECG, clinic or cardioangiography were not complete; AMI caused by coronary flow interruption due to invasive diagnosis and treatment, or other diseases instead of atherosclerosis.

### 2.2 Information collection

The following information was collected from the clinical records of the patients: baseline data of the cases: sex, age, disease history (diabetes, high blood pressure, high cholesterol), smoking; laboratory tests: taking venous blood within 24 hours of admission to detect the peak of cardiac muscle creatine kinase isozyme (MB iso-enzyme of creatine kinases, CK-MB), coronary angiography (CAG), IRA judgment, and percutaneous coronary intervention (PCI).

### 2.3 Coronary angiography and intervention treatment

All patients were taken to diagnostic emergency CAG via a standard right femoral artery path (Judkins technique) or right radial artery path. Two experienced cardiologists were independently responsible for the interpretation of IRA. The coronary angiography was done according to the previous descriptions.^[22]^ In briefly, only the patients with one occluded coronary artery were recruited. When the coronary artery recanalizes, the narrowest one is selected according to coronary angiography. We also had to see if there was filling defect or contrast agents retention in the narrow part. Standard techniques were used for PCI. All patients before intervention therapy were required chewing 300 mg aspirin and 300 mg clopidogrel. Intervention strategies, stents technology, selection of stents, and the use of hydrochloric were determined by the operator. Intervention treatment success: residual stenosis after stent implantation was less than 25% and forward flow was TIMI 3. After operation, all patients should take 100 mg of aspirin and 75 mg of clopidogrel one time a day for at least 12 months. All patients’ stents were drug-coated stents (DES).

### 2.4 Electrocardiogram (ECG)

Patients received electrocardiogram inspection immediately after admission or in the emergency room, with the paper speed being 25 mm/s and the standard voltage being 1 mm = 0.1 mV. The T-P was chosen as an equipotential lines. ST segment elevation: J point represented the ST segment, 80 ms after the J point was the measurement point of ST segment depression, ST segment depression ≥0.05 mV in a horizontal or oblique way was considered significant. Two cardiology attending physicians who were blind to the coronary angiography results separately analyzed the electrocardiographic tracings. If the results were inconsistent, the superior doctors were responsible for unifying the results.

### 2.5 Statistical analysis

The continuous variables were shown in 
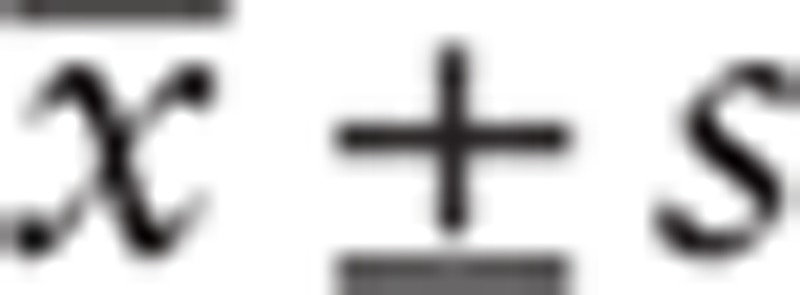
, and analyzed using student *t* test. *χ*^2^ or Fisher exact test were used for the analysis of the classified variables which were expressed as percentages. Predictive value of related lead electrocardiogram (ECG) on the LCX occlusion was analyzed according to the receiver-operating characteristic curve (ROC). All statistical analyses were performed using SPSS13.0 software (SPSS Inc., Chicago, IL). *P* values less than .05 were considered statistically significant.

## 3 Results

### 3.1 Baseline characteristics of the study population

According to coronary angiography, RCA was responsible for AIMI in 177 patients, while LCX was shown to be the IRA in 63 patients. The detailed information of the 2 groups was listed in Table 1. The demographic characteristics such as age and sex were similar between the 2 groups (*P* > .05 for both). The risk factors for CHD, such as hypertension, diabetes, dyslipdemia, smoke, and CK-MB, were compared between RCA and LCX groups. The results suggested that the occurrence of the related risk factors did not show significant difference between the 2 study groups (*P* > .05 for all).

**Table 1:**
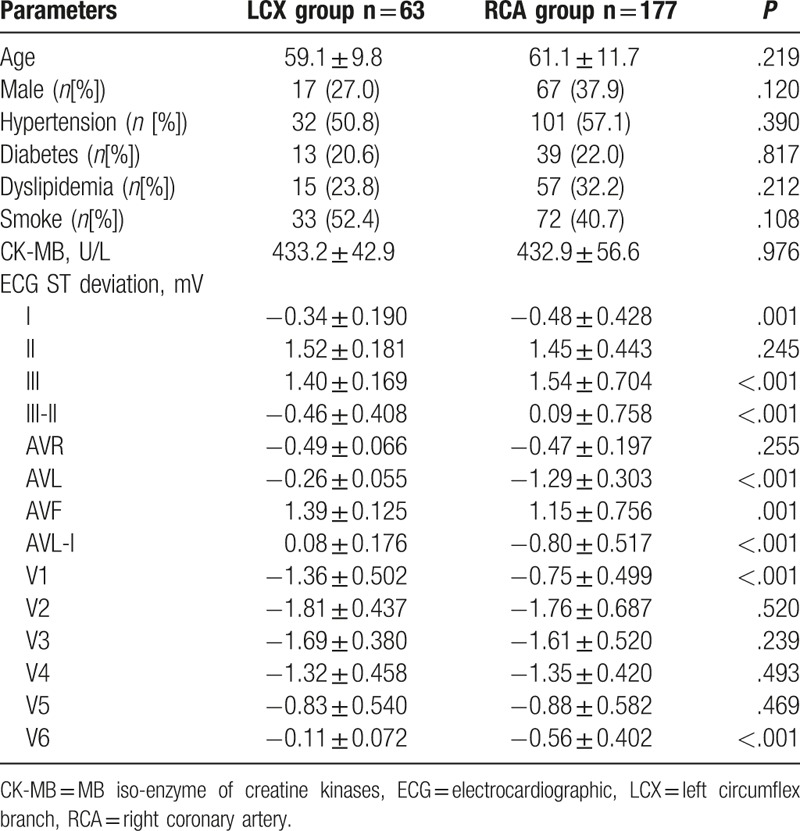
Baseline clinical date.

In addition, we also compared the electrocardiographic tracings between the 2 study groups. Analysis results demonstrated that ST-segment deviation, I, III–II, III, AVL, AVF, AVL-I, V1, V6 exhibited obvious difference between the 2 groups (*P* < .05 for all). However, ECG ST deviation, II, AVR, V2, V3, V4, and V5 showed similar between the 2 groups (all *P* > .05) (Table 1).

### 3.2 The predictive value of electrocardiogram on LCX occlusion

**Figure 1:**
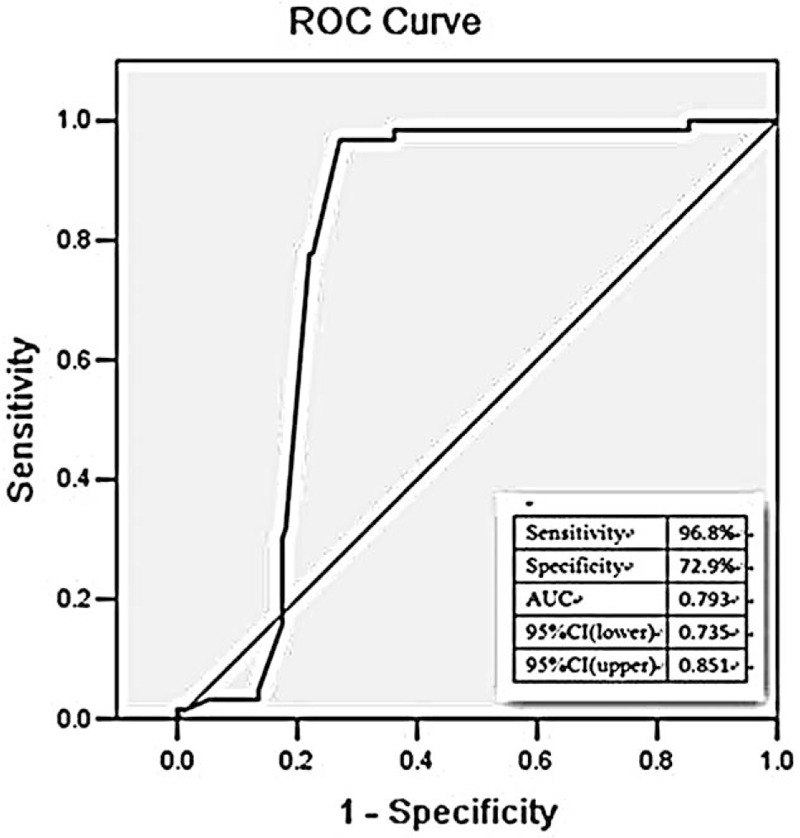
The relationship between STD in lead I and LCX of IRA in patients with AIMI. AIMI = acute inferior myocardial infarction, IRA = infarct-related artery, LCX = left circumflex branch, STD = ST segment depression.

**Figure 2:**
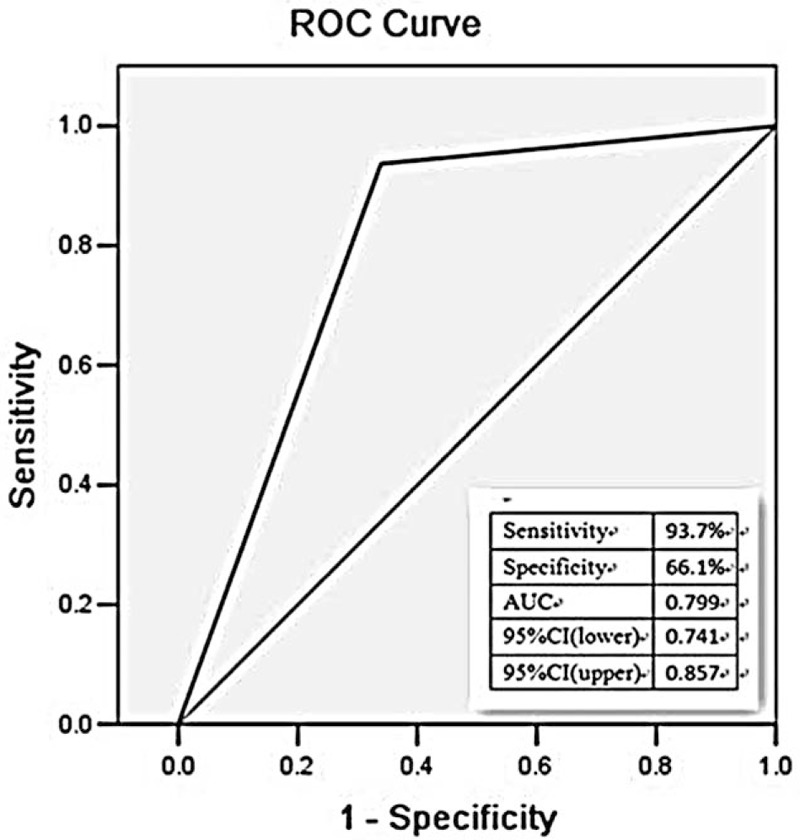
The relationship between STE in lead III < II and LCX of IRA in patients with AIMI. AIMI = acute inferior myocardial infarction, IRA = infarct-related artery, LCX = left circumflex branch, STE = ST segment elevation.

**Figure 3:**
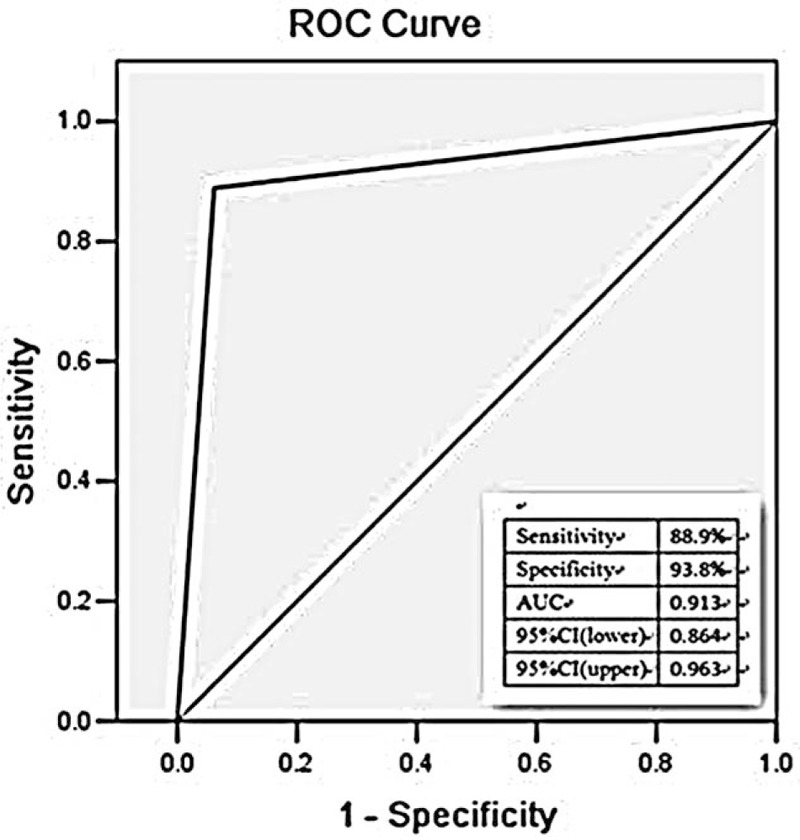
The relationship between STD in lead AVL < I and LCX of IRA in patients with AIMI. AIMI = acute inferior myocardial infarction, IRA = infarct-related artery, LCX = left circumflex branch, STD = ST segment depression.

In the current study, ROC analysis was performed to evaluate the diagnostic value of ECG in AIMI caused by LCX. The ROC curves were plotted based on the data of ST segment in patients in LCX and RCA groups. Among the study population, the diagnostic sensitivity and specificity of I lead ST segment depression (STD) on LCX occlusion were 96.8% and 72.9%, respectively, with the area under the ROC curve (AUC) of 0.793 (95% CI = 0.735–0.851) (Fig. 1). As showed in the Fig. 2, ST segment elevation (STE) lead III < lead II might be used as a diagnostic indicator for LCX occlusion, with the AUC value of 0.799 (95% CI = 0.741–0.857), combing with the sensitivity and specificity of 93.7% and 66.1%, respectively. We also investigated the diagnostic value of STD in AVL < I. ROC curve demonstrated that the AUC value was 0.913 (95% CI = 0.864–0.963), suggesting its capacity to act as a diagnostic biomarker for LCX occlusion. The diagnostic sensitivity was 88.9%, and the specificity was 93.8% (Fig. 3).

## 4 Discussion

Previous researches had showed that in acute inferior wall myocardial infarction, the proportion of RCA and LCX was roughly between 2.2:1 and 7.0:1, with an average of 3.9:1.^[25]^ This research showed that the proportion of RCA and LCX was 2.8:1, which was valuable for the diagnosis of STEMI caused by LCX occlusion combined multiple lead of ST segment deviation through the standard 12-lead electrocardiogram.

Chia et al^[26]^ study shows that ST segment elevation lead II > lead III or lead I ST segment having no depression is the diagnostic criteria of LCX occlusion. Chao et al^[27]^ study shows that when ST segment elevation (STE) lead III < lead II, lead AVL ST segment elevation, and lead AVR ST segment depression exist in the inferior wall myocardial infarction, the possibility of LCX occlusion increase. These 2 studies are both small sample research (more than 90 cases), and the target populations were all Asian people. They were divided into 2 groups, the right coronary artery group and left circumflex branch group. Circumflex branch lesions accounted for 22.0% and 30.6%, respectively, which suggested that the possibility of LCX occlusion increase when STE II > III, also, STD I, STE AVL, and STD AVR has some predictive value for LCX occlusion. The predictive sensitivity and specificity of STE II > III for LCX occlusion were 73.3% and 88.2%, respectively. The study sample was 240, 63 cases of cyclotron lesions accounting for 26.3%, which was similar to the proportion of inferior wall myocardial infarction in literature; the grouping situation was similar to that of literature; STD I (AUC = 0.79), STE II > III (AUC = 0.80), STE V6 (AUC = 0.85), STD AVL < I (AUC = 0.91) through ROC curve analysis in inferior wall myocardial infarction have some predictive value in LCX occlusion. The predictive sensitivity and specificity of STD I for LCX occlusion were 96.8% and 72.9%, respectively. The predictive sensitivity and specificity of STE II > III for LCX occlusion were 93.7% (above 73.3% in literature) and 73.3% (less than 88.2% in literature), respectively, which was similar to Chia study results. The possible mechanism may be as follows: when the occlusion of the left circumflex branch occurs, ST vector points in the direction of the back side results in STE II > III. The predictive sensitivity and specificity of STE V6 for LCX occlusion were 76.2% and 93.2%, respectively. Noriega et al^[28]^ research showed that the predictive sensitivity and specificity of STE V6 for LCX occlusion were 71.0% and 83%, respectively. The results of our study did not show STE V6, which may result from the small sample size of LCX occlusion. The predictive sensitivity and specificity of STD AVL < I for LCX occlusion were 88.9% and 93.8%, respectively, with the former slightly better than the latter. Possible advantages were as follows: when left circumflex branch occluded, ST vector to the left side with an angle with lead AVL. As a result, ranges of ST segment change were smaller than the right coronary occlusion, and occlusion of the left circumflex branch can cause sidewall ischemic damage at the same time and side wall lead ST segment elevation. These two changes offset each other, and ECG showed that the range of STD AVL was lower than that of I lead, even elevated. The changes of ST segment of lead AVR had no statistical significance in 2 groups, which was not consistent with the results of Kanei et al^[29]^ study, which suggested STD AVR had some predictive value for LCX occlusion.

### 4.1 Study limitations

Although we proved the diagnostic value of ECG in IRA identification, there were still several limitations in the current study. First, as a single-center retrospective study, all selected cases were patients with acute inferior wall myocardial infarction admitted to our hospital, the selection bias might influence the final results. Furthermore, emergency death and patients who refused to accept the treatment were not included that might also increase the bias in the current study. Second, the analysis results might be limited by the relatively small sample size in the present research, and further investigations with larger sample size were required to identify the clinical significance of ECG in determination of IRA for AIMI patients.

## 5 Conclusion

I lead ST segment depression, II lead ST segment elevation > III lead, AVL lead ST segment depression < I lead exhibit predictive value for the occlusion of cyclotron branches, which can be used for identification IRA in patients with AIMI. This new method is simple, practical, and noninvasive, especially important for medical institutions that cannot carry out the coronary artery imaging technology and patients who are unwilling to undergo coronary angiography examination.
